# *Capparis Spinosa L.* promotes anti-inflammatory response in vitro through the control of cytokine gene expression in human peripheral blood mononuclear cells

**DOI:** 10.1186/s12865-016-0164-x

**Published:** 2016-08-02

**Authors:** Mouna Moutia, Khadija El Azhary, Anass Elouaddari, Abdellah Al Jahid, Jamal Jamal Eddine, Fouad Seghrouchni, Norddine Habti, Abdallah Badou

**Affiliations:** 1 Laboratory of Hematology and Cellular and Genetic engineering, Faculty of Medicine and Pharmacy, Hassan II University, Casablanca, Morocco; 2 Laboratory of Experimental Medicine and Biotechnology, Faculty of Medicine and Pharmacy, Hassan II University, Casablanca, Morocco; 3 Research team Health and Environment, Cadi Ayyad University, Polydisciplinary Faculty, Safi, Morocco; 4 Laboratory of Synthesis, Extraction and Physicochemical study of organic molecules, Faculty of Sciences Ain Chock, Hassan II University, Casablanca, Morocco; 5 Laboratory of Cellular Immunology, National Institute of Hygiene, Rabat, Morocco; 6 Present Address: Cellular and Molecular Pathology Laboratory, Faculty of Medicine and Pharmacy, Hassan II University, Casablanca, Morocco

**Keywords:** *Capparis Spinosa*, Peripheral blood mononuclear cells, Anti-inflammation, Cytokines, Gene expression, Cell proliferation, Cell viability

## Abstract

**Background:**

*Capparis Spinosa* L. is an aromatic plant growing wild in dry regions around the Mediterranean basin. *Capparis Spinosa* was shown to possess several properties such as antioxidant, antifungal, and anti-hepatotoxic actions. In this work, we aimed to evaluate immunomodulatory properties of *Capparis Spinosa* leaf extracts in vitro on human peripheral blood mononuclear cells (PBMCs) from healthy individuals.

**Results:**

Using MTT assay, we identified a range of *Capparis Spinosa* doses, which were not toxic. Unexpectedly, we found out that *Capparis Spinosa* aqueous fraction exhibited an increase in cell metabolic activity, even though similar doses did not affect cell proliferation as shown by CFSE. Interestingly, *Capparis Spinosa* aqueous fraction appeared to induce an overall anti-inflammatory response through significant inhibition of IL-17 and induction of IL-4 gene expression when PBMCs were treated with the non toxic doses of 100 and/or 500 μg/ml. Phytoscreening analysis of the used *Capparis Spinosa* preparations showed that these contain tannins; sterols, alkaloids; polyphenols and flavonoids. Surprisingly, quantification assays showed that our *Capparis Spinosa* preparation contains low amounts of polyphenols relative to *Capparis Spinosa* used in other studies. This *Capparis Spinosa* also appeared to act as a weaker scavenging free radical agent as evidenced by DPPH radical scavenging test. Finally, polyphenolic compounds including catechin, caffeic acid, syringic acid, rutin and ferulic acid were identified by HPLC, in the *Capparis spinosa* preparation.

**Conclusion:**

Altogether, these findings suggest that our *Capparis Spinosa* preparation contains interesting compounds, which could be used to suppress IL-17 and to enhance IL-4 gene expression in certain inflammatory situations. Other studies are underway in order to identify the compound(s) underlying this effect.

**Electronic supplementary material:**

The online version of this article (doi:10.1186/s12865-016-0164-x) contains supplementary material, which is available to authorized users.

## Background

Among Peripheral Blood Mononuclear Cells (PBMCs), there are Monocytes, Natural Killer cells, B cells and T cells. Naïve CD4+ T cells can differentiate into several distinct subpopulations including Th1, Th2 and Th17 cells. Identification of these T cells subsets is based mainly on their immunological functions that are supported by the specific cytokines these different cells produce. Th1 cells are characterized by high secretion of IFN-γ and IL-2. These cells are responsible for intracellular pathogen elimination [1]; but are also involved in the development of organ-specific autoimmune diseases, as well as chronic inflammatory disorders [2]. Th2 cells are implicated in humoral immunity and provide protection against parasites. They also play a major role in the initiation, maintenance, and amplification of human allergic inflammation [3]. Th2 cells produce IL-4, IL-10 and IL-13, which are involved in allergic asthma [3, 4]. Th17 cells produce mainly IL-17A and IL-17F and take a major part in the clearance of extracellular bacteria and fungi, due to their capacity to recruit and activate Neutrophils [5]. In contrast, they promote the pathogenesis of cancer [6], several autoimmune and inflammatory diseases [7], such as multiple sclerosis, rheumatoid arthritis, inflammatory bowel disease, psoriasis and contact dermatitis [8, 9]. New compounds could have a great interest, in several pathophysiological situations such as autoimmunity [7] and cancer [6], if they are able to suppress the expression of pro-inflammatory cytokines such as IL-17. These molecules could be synthetic or natural, extracted from plants.

*Capparis Spinosa L*. (C.S.) is an aromatic plant growing wild in dry regions around the Mediterranean basin [10, 11]. Different parts of this plant, including the flower buds, fruits, seeds, and roots, were traditionally used in folk medicine to treat rheumatism, digestive problems, headaches, and toothaches. C.S. was also used as a diuretic, antihypertensive and tonic [10, 12, 13]. It has also been shown that C.S. extracts possess several bioactivities such as antioxidant, antifungal, anti-hepatotoxic and anti-inflammatory actions [14, 15]. Reports showed that C.S. contains alkaloids, lipids, polyphenols, flavonoids, indole and aliphatic glucosinolates [16, 17]; which are known to possess bioactive properties.

Specific T cell subsets could be stimulated to produce specific cytokines after their interaction with different natural or synthetic molecules and cytokines [18–20]. Their specific cytokines initiate and orientate the immune response. On this basis, they are divided into pro-inflammatory (e.g., IL-17, INF-γ, TNF-α) and anti-inflammatory (e.g., IL-4, IL-10, TGF-β) cytokines. Here, we aimed to evaluate potential anti-inflammatory properties of C.S. leaves on human peripheral blood mononuclear cells. Our data indicate that, doses of C.S. extracts, which are not toxic and do not affect cell proliferation, are able to suppress pro-inflammatory cytokines such as IL-17 and promote expression of anti-inflammatory cytokines such as IL-4. C.S. may therefore contain compounds that could be used as anti-inflammatory in certain pathophysiological situations. Studies are in progress in order to identify these compounds.

## Results

### Cell viability and metabolic activity upon treatment with *Capparis Spinosa*

**Fig. 1:**
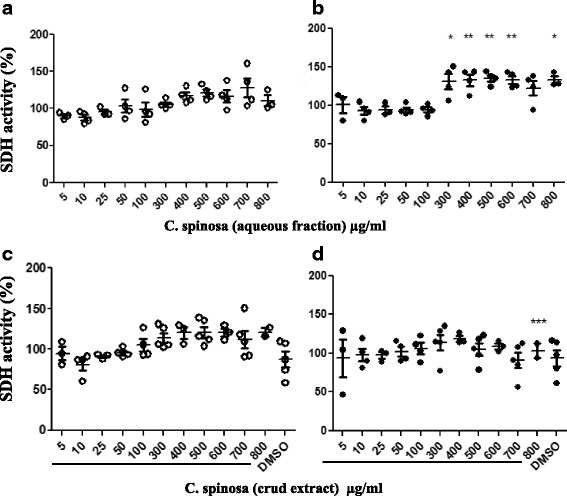
Increasing doses of *Capparis Spinosa* aqueous fraction enhanced MTT reduction in human PBMCs. Human PBMCs were cultured for 4 days with or without PHA (5 μg/ml) in the presence of increasing doses of the aqueous fraction or the crud extract of *Capparis Spinosa*, Panel **a**: PBMCs without PHA in the presence of different doses of the aqueous phase of C.S. Panel **b**: PBMCs stimulated with PHA in the presence of different doses of the aqueous fraction of C.S. Panel **c**: PBMCs without PHA in the presence of different doses of the crude extract of C.S. Panel **d**: PBMCs stimulated with PHA in the presence of different doses of the crud extract of C.S. Three to five independent experiences were performed. Data were analyzed using the one-way ANOVA test. (*, **, ***) indicate a *P* value of less than 0.05; 0.01 and 0.001 respectively

In order to test whether C.S. preparations could contain compounds that have the ability to regulate immune system cells and eventually suppress pro-inflammatory cytokines, we started by assessing cell toxicity. Human PBMCs were incubated with increasing doses of either C.S. crud extract or the corresponding aqueous fraction, in the presence or absence of phytohaemagglutinin (PHA). Our results showed that both C.S. preparations did not induce cell toxicity, even when used at relatively high doses (700 μg/ml) (Fig. 1a and c). This absence of toxicity was observed also when the PHA was added (Fig. 1 b and d). Interestingly, we noticed unexpected and significant increase in succinat deshydrogenase (SDH) enzymatic activity upon PHA stimulation in the presence of the aqueous (Fig. 1b) but not the crud (Fig. 1d) extract of C.S. This enhancement of the enzymatic activity of mitochondrial SDH may reflect an increase in proliferation and/or other cell function as reported in previous papers [21, 22]. This effect was more pronounced in the presence of the aqueous fraction relative to the crud extract (Fig. 1b and d) and was statistically significant (Fig. 1b, and Additional file 1).

### Cell proliferation upon treatment with *Capparis Spinosa*

**Fig. 2:**
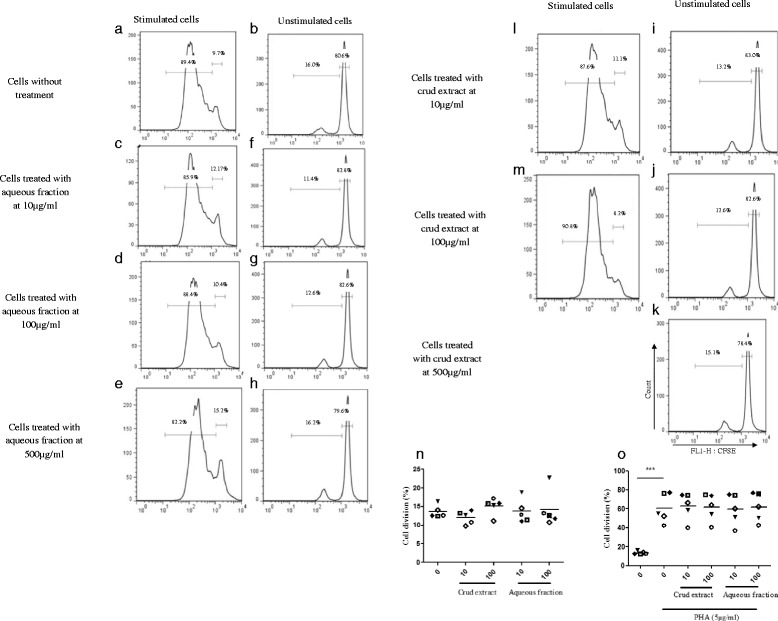
*Capparis Spinosa* crud extract and aqueous fraction did not affect proliferation of human PBMCs. **a** CFSE-stained PBMCs stimulated with PHA 5 μg/ml. **b** CFSE-stained PBMCs in the absence of stimulation. **c**, **d**, and **e** an example of curves generated by CFSE-stained PBMC, stimulated with PHA 5 μg/ml and harvested with 10, 100 and 500 μg/ml respectively of aqueous fraction, **f**, **g** and **h** an example of curves generated by CFSE-stained PBMC harvested with of 10, 100 and 500 μg/ml of aqueous fraction, for 4 days. **i**, **j** and **k** An example of curves generated by proliferation of CFSE-stained PBMC harvested with 10, 100 and 500 μg/ml of crud extract for 4 days of culture. **l** and **m** An example of curves generated by CFSE-stained and stimulated PBMC in the presence of 10 and 100 μg/ml respectively of the crud extract, for 4 days. **n** and **o** Cumulative data from five independent experiments analyzed statistically using one-way ANOVA test. (***) indicate a *P* value of less 0.001 respectively. Each peak represents a cycle cell division. The curves generated by the CFSE profile were analyzed using the proliferation platform of the FlowJo software. Data shown represent results of five independent experiments

Since C.S. aqueous fraction showed enhanced SDH enzymatic activity on PBMCs, we tested whether this could be related to an enhancement of cell proliferation. To assess this hypothesis, we used CFSE and flow cytometry. CFSE-labeled PBMCs were stimulated with PHA in the presence or absence of different doses of C.S. preparations (crud extract or aqueous fraction) for 96 h. While CFSE-labeled, PHA-treated PBMCs showed marked proliferation compared to control cells (Fig. 2a, b, and o), neither of the two extracts of C.S. induced proliferation either alone (Fig. 2f, g, h, i, j, k and n) or in the presence of PHA (Fig. 2c, d, e, l, m and o, and Additional file 2).

### Qualitative phytochemical analysis and antioxidant activity of *Capparis Spinosa*

In order to identify potential bio-active chemical classes present in C.S. extracts, we proceeded to a qualitative chemical screening. As depicted in Table 1, we found that our extract contained: tannins, sterols, alkaloids, polyphenols and flavonoids. Subsequently, we quantified total polyphenols and total flavonoids in C.S. extract and found about 0.08247 mg galic acid equivalent /g of dried extract; and about 0.02129 mg quercitin equivalent /g of dried extract (Table 2), total flavonoid represents 25.8 % of total polyphenolic compounds.

**Table 1:** Phytochemical analysis of *Capparis Spinosa*’s crud extract and aqueous fra*ction*

Chemical class	Crud extract and aqueous fraction
Total flavonoids	+
Flavon/glycone	+
Sterols	+
Tannins	+
Alkaloïds	+
Saponosids	-
Triterpens	-

- = Negative result, + = Positive result

**Table 2:** Dosage of polyphenols and flavonoids in *Capparis Spinosa* crud extract

Concentration (Mean+ /-SD)	Crud extract of *Capparis Spinosa*
Polyphenols (mg galic acid equivalent/g)	0,08247+/− 0,001201
Flavonoïds (mg quercitin equivalent/g)	0,02129 +/− 0,0001626

**Fig. 3:**
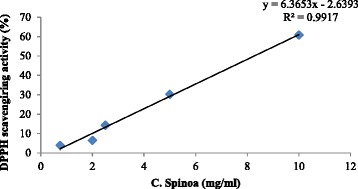
*Capparis spinosa*’s low anti-oxidant activity by DPPH Radical Scavenging test

On the other hand, antioxidant activity was assessed using DPPH radical scavenging test. The 50 % inhibition concentration (IC50) of scavenging free radical effect was evaluated and found to be 8.27 mg of C.S. crud extract per ml. This value was far lower than that obtained with ascorbic acid, used as positive control, known by its high antioxidant activity (IC50 = 0.100 +/− 0.004 mg/ml for DPPH) (Fig. 3, and Additional file 3).

**Fig. 4:**
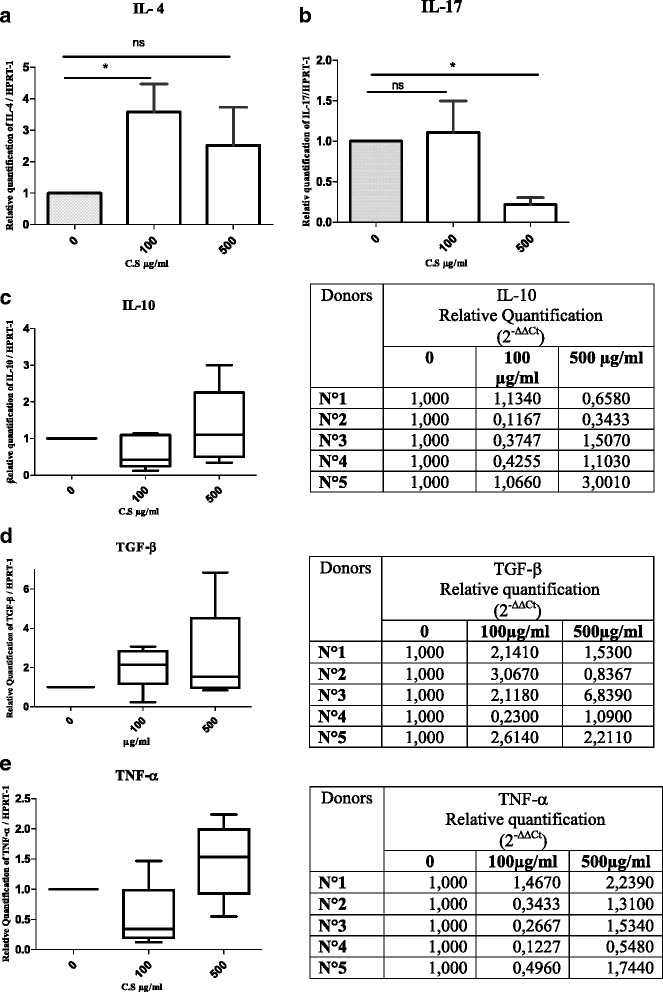
Effects of *Capparis Spinosa*’s aqueous fraction on IL-4, IL-17, IL-10, TGF-β and TNF-α expression in PBMCs in culture. **a** IL-4, **b** IL-17, **c** IL-10, **d** TGF-β, **e** TNF − α. Incubated for 18 h, doses used 100 and 500 μg/ml. Data represent the mean ± S.D. Data from *n* = 5 separate experiments. Data were analyzed using the one-way ANOVA (Kruskal-Wallis test), flowed by Dunn’s post test (*) indicate a *P* value of less than 0.05

The effect of C.S. on pro and anti-inflammatory cytokines was then evaluated using doses of C.S., which were found to be not toxic. Figure 4 shows the effect of C.S. aqueous fraction on the expression of 5 cytokines after 18 h treatment. Data in panel A shows increases in IL-4 gene expression in cells treated with the aqueous fraction of C.S. This increase was, however, statistically significant with 100 μg/ml but not with 500 μg/ml of the aqueous fraction of C.S. This observation suggests that C.S. promotes induction of the IL-4 known as anti-inflammatory Th2 cytokine. In Panel B, we found a clear decrease in IL-17 gene expression when PBMCs were treated with 500 μg/ml of C.S. aqueous fraction. This decrease was statistically significant. In contrast, cells treated with 100 μg/ml did not show any significant effect on IL-17 gene expression. These data suggested that C.S. acts as an anti-inflammatory factor. Indeed, C.S. consistently promoted IL-4 (an anti-inflammatory cytokine) and inhibited IL-17 (a pro-inflammatory Th17 cytokine). Regarding IL-10, we did not detect any significant effect on PBMCs treated with either 100 or 500 μg/ml of the aqueous fraction of C.S. (Fig. 4 panel c). In fact, we observed a tendency of decrease using 100 μg/ml, and a tendency of increase at 500 μg/ml of the aqueous fraction of C.S. However, none of these effects were statistically significant (Fig. 4 panel c). Tendencies of increases in TGF-β with both 100 and 500 μg/ml and decreases in TNF-α were detected. However, these effects were not statistically significant (Fig. 4, panel d and e, and Additional file 4). We also tried to quantify the expression of IFN-γ in our experiments, but we could detect its expression in only one out of five tested donors; thus no conclusions could be drawn. When PBMCs were stimulated with PHA and incubated with C.S. aqueous fraction (Additional file 5: Figure S1, Panels G, H, I, K and J), we did not detect any significant effect in C.S.- treated relative to control cells for any of the cytokines tested, IL-4, IL-17, IL-10, TGF-β and TNF-α. Altogether our data suggest that 100 μg/ml of C.S. aqueous fraction is efficient and sufficient to affect cytokine gene expression, by stimulating the expression of anti-inflammatory cytokines namely IL-4 and TGF-β and inhibiting the expression of pro-inflammatory cytokine TNF-α. On the other hand, C.S. aqueous fraction at 500 μg/ml showed a clear inhibition of another pro-inflammatory cytokine, IL-17. These observations indicate that the C.S. aqueous fraction may contain compounds endowed with anti-inflammatory properties.

### Analysis of *Capparis Spinosa* extract by HPLC

**Fig. 5:**
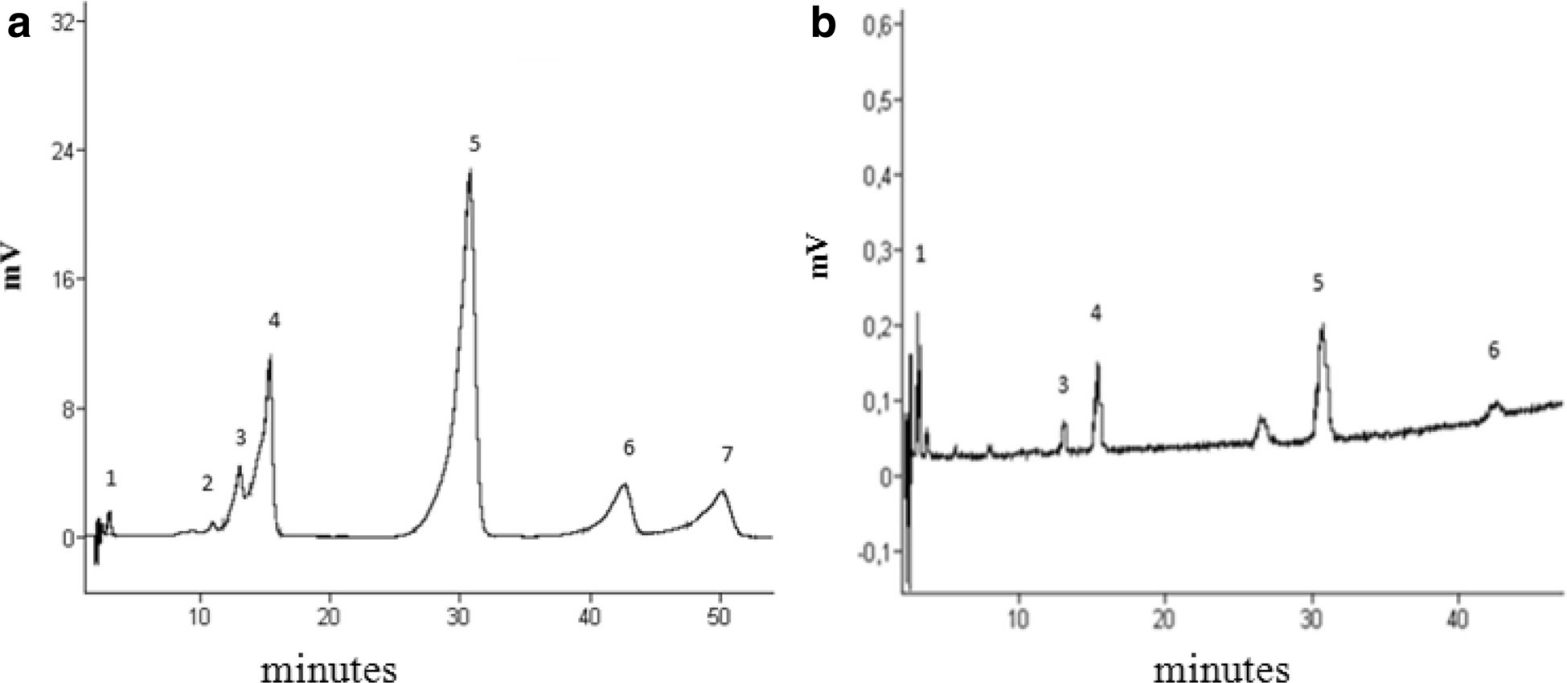
Identification of several polyphenol and flavinoid compounds in *Capparis Spinosa* extract. **a** Chromatogram of 7 available polyphenol and flavonoid standards monitored at 280 nm and identified with retention times (minutes) 1: catechin, 2: vanillic acid, 3: caffeic acid, 4: syringic acid, 5: rutin, 6: ferulic acid, 7: vanillin. **b** HPLC chromatogram of of *Capparis Spinosa* extract obtained under optimum conditions: 88 % water and 12 % acetonitril, 50 min. Peak no. 1: catechin, 3: caffeic acid, 4: syringic acid, 5: rutin and 6: ferulic acid were identified

In order to identify several known polyphenol and flavonoid compounds present in our C.S. extract, we have analyzed it by HPLC at 285 nm, using a mixture of 7 standards, which are catechin, vanillic acid, caffeic acid, syringic acid, rutin, ferulic acid, and vanillin (Fig. 5a). Five compounds were identified from the extract by matching their retention times to those of the standards (Fig. 5b). These compounds are catechin, caffeic acid, syringic acid, rutin and ferulic acid. Peak assignment was confirmed by injection of standards.

## Discussion

Immunomodulation using medicinal plants (or their compounds) can provide alternatives to the conventional therapy for a variety of diseases [23]. It could be effective, for instance, in cases of immune deficiency, autoimmunity or cancer to appropriately regulate the immune response and maintain a disease free state. In this study, we aimed to decipher immunomodulatory effects of C.S. leaf extracts on human peripheral blood mononuclear cells using C.S. plant from Morocco. In order to test whether C.S. could exhibit potential anti-inflammatory or other beneficial immunomodulatory effects, non toxic doses of C.S. extracts were determined using MTT assay. Subsequently, these non toxic doses were used to evaluate the C.S. effect on cell proliferation using CFSE. Our data revealed that doses up to 800 μg/ml of C.S. preparations were not toxic to human PBMCs (Fig. 1). Unexpectedly, we detected an increase in SDH enzymatic activity using doses of the C.S. aqueous fraction ranging from 300 to 600 μg/ml (Fig. 1b). This suggested either enhanced proliferation and/or enhanced metabolic activity of cells upon treatment with C.S. Consistent with our data, similar range of doses from 400 to 600 μg/ml were also shown to be non cytotoxic when lyophilized extracts of Italian C.S. buds were used [24].

It has been demonstrated in previous studies, that C.S. extracts elicit a protective effect in pathological conditions related to the oxidative stress status [14, 25]. This effect is likely due to effective antioxidant/free radical scavenging properties [25]. Indeed, C.S. was shown to be rich in polyphenols, which are known to be effective antioxidant/free radical scavenging compounds [15, 25, 26]. Therefore, we tested whether our extracts, obtained from the Moroccan C.S. leaves, could show similar protective effects. In this aspect, we began with a qualitative screening that confirmed the presence of flavonoids and polyphenols. Then we quantified their presence, and found that our C.S. extract is not as rich in polyphenols as other C.S. extracts used in other studies and obtained from different region of the world [10, 13, 15, 25, 27, 28]. In fact, in our extract, total polyphenol content was 0.08247 mg GAE/g of dried extract which is far inferior to Tunisian and Indian C.S. [13, 29]. Comparing the total polyphenol content of our leaf extract to that of extracts from other parts of the plant (performed in previous studies), ours is much lower than Italian C.S. flower buds [15, 25], Tunisian flower buds [13] and lower than Bahrainian [28] and Turkish C.S. fruit [27]. On the other hand, in our C.S. extract, total flavonoid content was 0.02129 mg quercitin equivalent /g of dried leaf extract. In comparison to other C.S. that are considered as a rich source of flavonoids, our values appear to be lower than those of Bahrainian C.S. fruit [28] and those of Indian C.S. [29]. These variations and differences in total phenol and flavonoid contents might be explained by geographical factors and by distinct processing methods. We also compared the IC50 of the scavenging free radical test obtained from our study to those obtained in previous studies. The IC50 of our C.S. leaf extract was 8.27 mg/ml of crud extract, which is similar to the Bahrainian C.S. fruit [28], and far weaker than the Italian C.S. bud extracts (0.177 mg of extract/ml) [25]; 0.068 mg/ml [15, 25] and the Turkish Caper fruit extract (0.32 mg/ml) [27]. Our C.S. leaf extract appears also to be by far a weaker scavenging free radical agent relative to the Indian C.S. leaf extract (0.073 mg/ml) [29]. Worth specifying that direct comparison of data from the current study with those reported in the literature is difficult since different parts of the plant and different expression units were used. However, in light of these data, the low anti-oxidant activity of the Moroccan C.S. leaf crud extract might be due to low amounts of total polyphenolic and flavonoid compounds known as effective anti-oxidant molecules.

Cytokines are key elements of the innate and adaptive immune response and provide a window through which diseases can be monitored and eventually controlled [30]. So we focused on the effects of C.S. extract on cytokine expression. In our present study, we showed that the aqueous fraction of C.S. leaf extract seems to have a modulator effect on IL-4, IL-17 in PBMCs from healthy donors. This consists of a stimulation of IL-4 expression (anti-inflammatory cytokine) and an inhibition of IL-17 expression (pro-inflammatory cytokine). Additionally, we detected a tendency of stimulating TGF-β expression in parallel with a trend of inhibiting TNF − α expression in PBMCs treated with C.S. extract though these effects were not statistically significant in our experiments. Finally, we could not detect consistent expression of IFN-γ gene in human PBMCs even in the absence of treatment with C.S. extract. The induction of IL-4 and the inhibition of IL-17 by C.S. are interesting since these are anti and pro-inflammatory cytokines, respectively. IL-4 is produced mainly by Th2 cells (humoral immunity) and is a primary driver of the Th2 phenotype; it is involved in down regulation of inflammatory and Th1-mediated responses [31, 32]. On the other hand, IL-17 is the hallmark of Th17 cells. It is considered a major pro-inflammatory cytokine, with pivotal roles in inflammatory and autoimmune diseases including Rheumatoid Arthritis and Multiple Sclerosis [33–35]. These data suggest an overall anti-inflammatory effect of C.S. on the immune response. Compounds from this plant could eventually play, in the future, an interesting role in modulating several pathologies in which IL-17 plays an important task. For instance, an involvement of IL-17 was also strongly suggested in tumor development and in both inflammation and sporadic cancers of the liver, stomach, and colon [36]. In the same time, the interesting trends observed in our work; increased TGF-β expression and decreased TNF-α expression in cells treated with C.S., are consistent with the modulatory effect observed on IL-4 and IL-17. Indeed, TGF-β is mostly assigned as an anti-inflammatory cytokine (produced by Treg cells), which achieves distinct immunosuppressive properties on various aspects of the immune response and also maintain self tolerance [37, 38]. On the other hand, TNF-α is a major pro-inflammatory cytokine, which plays relevant roles in pathogenesis of many autoimmune diseases and stimulates secretion of other inflammatory cytokines [39]. High levels of TNF-α are indeed associated with various acute and chronic inflammatory conditions [40, 41]. Our data seem to be different from those published in another report [24] in which C.S. extract failed to induce IL-4 production and rather up-regulated, pro-inflammatory cytokines (TNF-α and IFN-γ) in human PBMCs. These effects were attributed to the richness of Italian C.S. in polyphenols and flavonoids. In our experiments, the tendency of inhibiting TNF-α expression might be due either to a direct effect of extract compounds on the signaling cascade leading to TNF-α expression or to IL-4 induction also known to inhibit TNF-α [42]. TGF-β, which is an important anti-inflammatory cytokine, known to antagonize TNF-α and IFN-γ, was also stimulated by C.S. The overall inhibition of IL-17 and of TNF-α in addition to the stimulation of IL-4 and TGF-β implies that there might be an enhancement of anti-inflammatory cytokine in the account of pro-inflammatory cytokines.

HPLC analysis of C.S. extract showed the presence of various bioactive polyphenolic and flavonoid compounds, known for their anticancer potential, anti-inflammatory and immunomodulatory effects. For instance, Catechin is a flavonoid and a major compound of the catechines family, which were famous for their ability to control lymphocyte proliferation and modulate IL-2 and IFN-γ secretion in human PBMCs and mouse splenocytes [43]. Data from a previous work showed that catechin exerts an inhibition of immune activation and a regulation of unbalanced levels of IL-17/IL-10 [44]. It reversed abnormal polarization of Th17 as well as Treg cells in rat peripheral blood and spleen, by oral administration. It suppressed chronic heart failure induced by abdominal aorta ligation, which was closely associated with catechin modulation of Th17 and Treg in rats. With regard to caffeic acid, a derivative of cinnamic acids which are part of phenolic acids, is structurally related to flavonoids and is a biologically active component. It has been found to possess anti-mitogenic, anti-inflammatory and immunomodulatory effects [45]. Caffeic acid inhibited colonic IL-17 expression and increased IL-4 expression [46]. It also decreased IL-1β production by 40 % and did not affect TNF-α and IL-6 in human blood [47]. As for syringic acid, which is from the same family as the caffeic acid, it was found to suppress significantly pro-inflammatory cytokines (IFN-γ, TNF-α and IL-6) in serum of mice suffering immune-mediated liver inflammation [48]. Ferulic acid was found to have a chemopreventive activity against colon and rectal cancer [49]. As for the compound rutin, also known as vitamin P, it is a very important plant phenolic compound because of its anti-inflammatory property [50]; [51]. Since it was found to be capable of suppressing increased IL-17, and enhancing decreased IL-4 expressions, a characteristic of dextran sulfate sodium (DSS)- induced colitis, and presented a partial protection against DSS-induced colitis in mice [52]. Rutin also, ameliorated TNBS-induced colitis in rats by inhibiting TNF-α-induced NF-kB activation [53]. Some reports also showed that rutin was effective on the chronic phase of rat adjuvant arthritis [54]. Rutin was found to possess, in addition to anti-inflammatory potential, an anti-carcinogenic effect [13, 55].

## Conclusion

The anti-inflammatory response obtained on human PBMCs with Moroccan C.S. preparations suggests a potential beneficial effect of compounds present in this plant in diseases, where these pro- and anti-inflammatory cytokines are involved such as Multiple Sclerosis, Rheumatoid Arthritis and Cancer. This anti-inflammatory effect might result from the direct effect of one or more of the identified compounds such as catechin or caffeic acid or from a synergistic interaction between different compounds present in our preparations, such as tannins, sterols, and alkaloids; or from a single bio-active molecule. At this point, our main focus is the identification of the compound(s) underlying this effect.

## Methods

### Capparis Spinosa

*Capparis Spinosa* leaves were collected from the region of Safi in Morocco (GPS coordination are 32°16 41N and 90° 07 56W, altitude 146.4), between August and September of 2013. It was identified by the help of a plant biologist in Biology department of the polydisciplinary faculty of Safi. A voucher (No. 93664) of plant specimen has been deposited in the herbarium of the Botanical Department of the Scientific Institute of Rabat, Morocco.

### Capparis Spinosa preparations

Leaves were washed, air-dried then incubated in methanol (Sigma, USA) for 48 h at room temperature. The methanol extract was filtered then concentrated by rotary evaporation. The obtained dried extract was divided into 2 parts. The first part was dissolved in DMSO (crud extract) (Sigma, USA) and the second part was dissolved in distilled water and agitated vigorously several times, filtered, concentrated using rotary evaporation and dissolved in distilled water (aqueous fraction). Crud extract and aqueous fraction were both stored at −20 °C until use.

### Phytochemical analysis

*Capparis Spinosa* aqueous fraction and crud extract were subjected to qualitative chemical screening for identification of various classes of active chemical constituents using a previously described method [56]. Screening was performed for Saponins, Tannins, Alkaloids, Sterols, triterpenes and flavone aglycones.

### Dosage of total phenolic compounds

Total phenols content in samples was determined by the Folin-Ciocalteau colorimetric method [57]. Extract (0.5 ml) was reacted with 2.5 ml of the Folin- Ciocalteau diluted ten times in distilled water for 4 min. Then, 2 ml of an aqueous solution of Na_2_CO_3_ (75 mg/ml) were added to the reaction mixture. After 2 h incubation at room temperature, the absorbance was measured at 760 nm. Gallic acid was used as a reference standard and total phenols content was expressed as mg gallic acid equivalents per gram of plant extract (GAE/g of dry weight). Experience was performed in triplicate.

### Dosage of total flavonoid compounds

The flavonoid content was measured according to a previously reported colorimetric assay [58]. To design the calibration curve, 1 ml of standard solution of quercetin prepared at different concentrations (0–25 μg/ml) was added to 1 ml of 2 % AlCl_3_ methanol solution. After incubation for 60 min at room temperature, the absorbance was read at 420 nm. The same reaction was applied to extract at different concentration (0.1–0.5 μg/ml). Total flavonoid content was expressed as mg quercetin equivalent per gram of plant extract. Samples were analyzed in triplicate.

### Antioxidant activity by DPPH radical scavenging test

In order to assess the antioxidant ability of the crud extract, the DPPH (2,2-Diphenyl-1-picrylhydrazyl) approach was used. This is based on the ability of the sample to reduce free radicals of a given color, which changes after the oxidation-reduction reaction. A more pronounced change indicated a stronger antioxidant activity. Quantification was performed by spectrophotometry. DPPH in the presence of an antioxidant lowers the carmine color intensity up to very pale yellow. Data are presented as IC50, which means the concentration of a sample that reduces oxidation to half (50 %). The DPPH radical scavenging activity was evaluated according to a previously described method [59]. Ethanolic solution of extract and DPPH (Sigma-Aldrich, Steinheim, Germany) were mixed in tube (25 μl: 975 μl). Tests were performed with different extract concentrations. After 30 min, measurement of the absorbance was performed at 517 nm at room temperature. Negative and positive controls were performed in the same conditions with ethanolic DPPH solution and Ascorbic acid, respectively. Tests were carried out in triplicates. The inhibition percentage of DPPH radical was calculated according to the following formula:

$$ \mathrm{Scavenging}\ \mathrm{effect}\ \% = \kern0.5em \left[\left(\mathrm{A}1\ \hbox{--}\ \mathrm{A}0\right)\ /\ \mathrm{A}0\right]\kern0.5em *\kern0.5em 100; $$

Where A0 was the absorbance of the control without extract and A1 was the absorbance of the sample. Sample concentration providing 50 % inhibition (IC50) was obtained by plotting the inhibition percentage against sample concentrations.

### Polyphenol and flavonoid compounds analysis by HPLC

Qualitative analysis of standard phenolic compounds in *Capparis Spinosa* extract was performed using a high performance liquid chromatography (HPLC) type JASCO PU-1580, equipped with a UV detector/Vis type JASCO 875 UV and a data treatment station Azur version 3.0.3.0. The extract was evaporated to dryness at 40 °C and then taken up with a mixture of distilled water - acetonitril (88–12 %). After vigorous stirring, the mixtures were filtered by passing the solution through nylon membrane Whattman. Analysis conditions used were: Column: C18 (1.7 μm 2,1 × 150 mm), UV Wavelength: 285 nm, 1 ml/min, Gradient elution program was set as water - acetonitrile (88–12 %). The solution of the polyphenol mixture: catechin, rutin, vanillin, vanillic acid, caffeic acid, syringic acid, and ferulic acid was prepared by dissolving 1–5 mg of each polyphenol in 1 ml of the acetonitril - water 12 −88 %. The final solution was filtered through a nylon membrane Whattman 0.22 μm.

### PBMC preparation and cell culture

Venous fresh blood was collected from fifteen healthy adult volunteers, aged between 18 and 45 years old, informed with written consent, among the fifteen donors, five donors were used for each type of assay, MTT, cell proliferation and RT-qPCR. PBMCs were isolated by centrifugation on Ficoll- histopaque (d = 1.077 g/ml) (Sigma, USA), at 900 g for 25 min at 18–20 °C. Cells were then washed three times in RPMI-1640 at 400 g for 15 min and re-suspended in RPMI-1640 media (sigma, USA) with L-glutamine, supplemented with 10 % heat inactivated newborn calf serum (sigma, USA), 26.3 g/l penicillin and 4.2 g/l streptomycin. Cells were counted using a hemocytometer and standard Trypan blue exclusion method and observed under microscopy. PBMCs were incubated at 37 °C with 5 % CO_2_ (Heracell 150i incubator CO2, thermosciences, France), in plat culture with different doses of plant extracts, with or without 5 μg/ml of phytohaemagglutinin (PHA; Sigma, USA). Tests were performed in triplicates.

### MTT assay

Cell viability was determined by the MTT (3-(4, 5 dimethylthiazol-2-yl)-2, 5 diphenyltetrazolium bromide) test [60]. Cells were cultured at 37 °C with 5 % CO_2_, in 96-well plates (Hiwaka, Japan) at a concentration of 5.10^5^ cells/ml, with various concentrations of the plant extract, in the presence or absence of PHA for 96 h. Enzymatic activity of each well was determined by MTT assay and compared to that of untreated cells. MTT (5 mg/ml, Sigma, USA) was dissolved in RPMI, filtered through a 0.2 μm filter and stored at - 20 °C. During the test, 20 μl of the MTT solution were added to each well and the corresponding plates were incubated at 37 °C, for 4 h in a humidified atmosphere with 5 % CO_2_. Subsequently, the plates were centrifuged at 800 g for 20 min, and 100 μl of DMSO were then added to each well, and mixed thoroughly to dissolve the crystals. High optical density readings corresponded to a high intensity of coloration, which is due to viable cells able to metabolize MTT salts. SDH enzymatic activity (%) was calculated using the following formula:

$$ \%\ \mathrm{of}\ \mathrm{S}\mathrm{D}\mathrm{H}\ \mathrm{enzymatic}\ \mathrm{activity} = \kern0.5em \left[\left(\mathrm{O}\mathrm{D}\ \mathrm{test}\hbox{-} \mathrm{O}\mathrm{D}\ \mathrm{control}\right)/\ \mathrm{O}\mathrm{D}\ \mathrm{control}\right]\kern0.5em *\kern0.5em 100. $$

### Cell proliferation assay

PBMCs were incubated in 96 well plates at a concentration of 10^6^ cells/ml. C.S. preparations were used at a final concentration of 10, 100 and 500 μg/ml, with or without PHA, in triplicate. PBMCs were labeled before culture with 5,6-carboxyfluorescein diacetate succinimidyl ester (CFSE) (Sigma, USA) at a concentration of 5 μM, according to a previous report [61]. Then, cells were washed twice with RPMI containing 5 % heat inactivated fetal calf serum. Cells were maintained in culture at 37 °C and 5 % CO_2_ for four days. Fluorescence measurements were performed using a FACS (BD FACSCalibur Flow Cytometer, BD Biosciences, France) and analyses were done using FlowJo software (Tree Star, Inc. Ashland, USA). One hundred thousand events were analyzed per sample.

### Gene expression quantification by RT-qPCR

#### Total RNA isolation and Reverse Transcription (RT)

Total RNA was isolated by Trizol (Sigma, France) from cells (10^6^ cells/ml) cultured alone or with different doses of C.S. preparations for 18 h in the presence or absence of PHA. Isolated RNAs were diluted in DEPC Treated Water (Invitrogene, France) and were measured with spectrophotometer (NanoVue™ Plus Spectrophotometer GE Healthcare UK Limited, UK). cDNA was synthesized using M-MLV reverse transcriptase (Reverse Transcriptase Super Scripte III, 10000 units, Invitrogene, France) from 0.5 μg total RNA, in a 20 μl reaction mixture according to the manufacturer’s instructions, with 1 μl of oligo dT20 (50 μM) and 1 μl of dNTP (10 mM of each) added and incubated at 65 °C for 5 min to break the secondary structure of RNA. Then the mixture was chilled on ice for at least 5 min. 4 μl of 5X Reverse Transcriptase buffer, 1 μl of RNase Inhibitor (RNasin OUT 5000 units, Invitrogene, France) and 1 μl of M-MLV reverse transcriptase were added and incubated at 50 °C for 60 min, then 70 °C for 15 min.

### Real-time qPCR assays

Relative quantification of gene expression was analyzed by real-time PCR using real time Fast 7500 (Applied Biosystems™). HPRT-1 was used as internal control. Amplifications were performed using SsoFast™ Eva Green® Supermix with Low ROX (BioRad, France) as recommended by the manufacture manual, in a 10 μl final volume, using primers at 500nM for all genes. PCR was programmed as follows: 30 s at 95 °C for polymerase activation and DNA sample denaturation; then 40 cycles of 15 s at 95 °C and 30 s at 60 °C for annealing and extension. Each reaction was performed in triplicate for each sample. The primers used are described in Table 3. Fluorescence readings at the end of the extension phase of each cycle were used to estimate the values for the threshold cycles (Ct). The Ct values for each gene were converted into relative quantification (2^-ΔΔCt^) using machine 7500 Software v2.0.6 software. The relative quantification for treated samples was calculated using cells with medium as a calibrator. PCR negative control (with no template) was included for each pair of primers.

**Table 3:** Primers sequences used for qPCR

Gene	Primer sequence	Transcript length (pb)
HPRT-1 Reverse Forward	GAGCACACAGAGGGCTACAA TGGACAGGACTGAACGTCTT	77
IL-4 Reverse Forward	GTTTTCCAACGTACTCTGGTTGGC ATGGGTCTCACCTCCCAACTGCT	506 357 405
IL-17 Reverse Forward	GTGGACAATCGGGGTGACAC ATCTCCACCGCAATGAGGAC	233
IL-10 Reverse Forward	GAAGCTTCTGTTGGCTCCC CTGTGAAAACAAGAGCAAGGC	500
TGF-β Reverse Forward	GCTGCACTTGCAGGAGCGCAC GCCCTGGACACCAATATTGC	336
TNF-α Reverse Forward	AAAGTAGACCTGCCCAGGACT ACAAGCCTGTAGCCCATGTT	427
IFN-γ Reverse Forward	CGACAGTTCAGCCATCAC GAAGAATTGGAAAGAGGA	265

### Statistical analysis

All data were expressed as means with standard deviations. Groups were compared using ANOVA-ONE way followed by post Bonferroni’s Multiple Comparison test and ANOVA-ONE way (Kruskal-Wallis test) flowed by Dunn’s post test, with a level of significance set at *p* < 0.05. Graphpad Prism 5.0 software was used for all statistical analyses.

## Abbreviations

C.S., *Capparis Spinosa*; CFSE, 5, 6-carboxyfluorescein diacetate succinimidyl ester; DPPH, 2, 2-Diphenyl-1-picrylhydrazyl, Ct: threshold cycles; MTT, 3-(4, 5 dimethylthiazol-2-yl)-2, 5 diphenyltetrazolium bromide; PBMCs, peripheral blood mononuclear cells; PHA, phytohaemagglutinin

## Additional files

Additional file 1: Raw data Figure 1. (XLSX 21 kb) Additional file 2: Raw data Figure 2. (XLSX 20 kb) Additional file 3: Raw data Figure 3. (XLSX 10 kb) Additional file 4: Raw data Figure 4 and supplementary figure. (XLSX 50 kb) Additional file 5: Figure S1. Effects of *Capparis Spinosa*’s aqueous fraction on IL-4, IL-17, IL-10, TGF-β and TNF-α expressions in stimulated PBMCs with PHA (5 μg/ml) in culture. (G) IL-4, (H) IL-17, (I) IL-10, (J) TGF-β, (K) TNF-α. incubated for 18 h, doses used 100 and 500 μg/ml. Data represent the mean ± S.D. Data from *n* = 5 separate experiments. (DOCX 108 kb)
