# Supplementary material for: Capparis Spinosa L. promotes anti-inflammatory response in vitro through the control of cytokine gene expression in human peripheral blood mononuclear cells
Source: BMC Immunol. 2016 Aug 2;17:26. doi: 10.1186/s12865-016-0164-x (PMC4969972; doi:10.1186/s12865-016-0164-x)
Supplement: Additional file 5: Figure S1. — Effects of Capparis Spinosa’s aqueous fraction on IL-4, IL-17, IL-10, TGF-β and TNF-α expressions in stimulated PBMCs with PHA (5 μg/ml) in culture. (G) IL-4, (H) IL-17, (I) IL-10, (J) TGF-β, (K) TNF-α. incubated for 18 h, doses used 100 and 500 μg/ml. Data represent the mean ± S.D. Data from n = 5 separate experiments. (DOCX 108 kb) [file 12865_2016_164_MOESM5_ESM.docx]

**Supplementary figure 1:**

**G H**

**I J**

K
